# Metabolic Syndrome Components Are Associated with Intervertebral Disc Degeneration: The Wakayama Spine Study

**DOI:** 10.1371/journal.pone.0147565

**Published:** 2016-02-03

**Authors:** Masatoshi Teraguchi, Noriko Yoshimura, Hiroshi Hashizume, Shigeyuki Muraki, Hiroshi Yamada, Hiroyuki Oka, Akihito Minamide, Yuyu Ishimoto, Keiji Nagata, Ryohei Kagotani, Sakae Tanaka, Hiroshi Kawaguchi, Kozo Nakamura, Toru Akune, Munehito Yoshida

**Affiliations:** 1 Department of Orthopaedic surgery, Wakayama Medical University, 811–1 Kimiidera, Wakayama, 641–8509, Japan; 2 Department of Joint Disease Research, 22^nd^ Century Medical & Research Center, Faculty of Medicine, The University of Tokyo, 7-3-1 Hongo, Bunkyo-ku, Tokyo, 113–8655, Japan; 3 Department of Medical Research and Management for Musculoskeletal Pain, 22nd Century Medical and Research Center, Faculty of Medicine, The University of Tokyo, 7-3-1 Hongo, Bunkyo-ku, Tokyo, 113–8655, Japan; 4 Department of Orthopaedic surgery, Faculty of Medicine, The University of Tokyo, 7-3-1 Hongo, Bunkyo-ku, Tokyo, 113–8655, Japan; 5 Japan Community Healthcare Organization Tokyo Shinjuku Medical Center, 5–1 Tsukudo-chome, Shinjuku-ku, Tokyo, 162–8543, Japan; 6 Rehabilitation Services Bureau, National Rehabilitation Center for Persons with Disabilities, 1 Namiki 4-chome, Tokorozawa City, Saitama, 359–8555, Japan; National Cancer Center, JAPAN

## Abstract

**Objective:**

The objective of the present study was to examine the associations between metabolic syndrome (MS) components, such as overweight (OW), hypertension (HT), dyslipidemia (DL), and impaired glucose tolerance (IGT), and intervertebral disc degeneration (DD).

**Design:**

The present study included 928 participants (308 men, 620 women) of the 1,011 participants in the Wakayama Spine Study. DD on magnetic resonance imaging was classified according to the Pfirrmann system. OW, HT, DL, and IGT were assessed using the criteria of the Examination Committee of Criteria for MS in Japan.

**Results:**

Multivariable logistic regression analysis revealed that OW was significantly associated with cervical, thoracic, and lumbar DD (cervical: odds ratio [OR], 1.28; 95% confidence interval [CI], 0.92–1.78; thoracic: OR, 1.75; 95% CI, 1.24–2.51; lumbar: OR, 1.87; 95% CI, 1.06–3.48). HT and IGT were significantly associated with thoracic DD (HT: OR, 1.54; 95% CI, 1.09–2.18; IGT: OR, 1.65; 95% CI, 1.12–2.48). Furthermore, subjects with 1 or more MS components had a higher OR for thoracic DD compared with those without MS components (vs. no component; 1 component: OR, 1.58; 95% CI, 1.03–2.42; 2 components: OR, 2.60; 95% CI, 1.62–4.20; ≥3 components: OR, 2.62; 95% CI, 1.42–5.00).

**Conclusion:**

MS components were significantly associated with thoracic DD. Furthermore, accumulation of MS components significantly increased the OR for thoracic DD. These findings support the need for further studies of the effects of metabolic abnormality on DD.

## Introduction

Intervertebral disc degeneration (DD) is generally considered as the first step of spinal change and undergoes destructive changes with age. It is typically followed by the loss of water and proteoglycan content of the nucleus, annulus tears, gradual formation of osteophytes, disc narrowing, and spinal canal stenosis [1, 2], and low back pain [3–6], is a major public health problem that negatively influences activities of daily living and quality of life in those affected. The number of patients with degenerative disease of the spine is increasing [6], thereby causing medical expenses to rise. In spite of these situation, the cause of DD is not fully understood. Because the etiology of DD exclude aging remains poorly understood. Accordingly, we need to clarify which risk factors promote DD to establish preventive measures against DD. In the present study, we focused on metabolic syndrome (MS) component, such as overweight (OW), hypertension (HT), dyslipidemia (DL), and impaired glucose tolerance (IGT), because MS component has some influence on atherosclerosis [7] and accumulation of MS component increase the risk of atherosclerosis events [8]. MS may increase not only the risk of cardiovascular events but also the risk of DD in the whole body [9], because intervertebral discs, which are structures with precarious nutrient supply at tissue level throughout the whole body, may suffer and gradually degenerate as a consequence of failure of nutrient supply to disc cells [10, 11]. However the association between MS component and DD remains controversial [9]. In some previous epidemiologic studies, OW [6, 12–16], DL [16], and IGT [17] were found to be associated with DD in the lumbar region. Other studies, however, have found no clear associations between hypertension (HT) [9], IGT [9, 16], and DL [9, 18], and DD in the lumbar region. This may be due to the limitation of potential biases related to patient selection and the consequences of disease on behavior. Furthermore, the majority of epidemiologic investigations have focused only on the lumbar spine. We believe that analysis of DD in the entire spine would provide more useful data than that of DD in only the lumbar region. Since the cervical and lumbar regions comprise mobile segments, the intervertebral discs in these regions are easily affected by mechanical and motion stress; thus, the effects of certain factors imposed on all intervertebral discs equally, such as age and endogenic factors, might be masked. In contrast, the thoracic region is stabilized by the thoracic cage, which reduces mechanical stress imposed on the intervertebral discs. We conducted a thorough literature review and found no studies of associations between component of MS and DD that focused on a population-based analysis using whole-spine magnetic resonance imaging (MRI).

The purpose of the present study was to examine the association of each MS component, such as OW, HT, DL, and IGT, with DD in the cervical, thoracic, and lumbar regions of the entire spine in a large population. We also examined the relationship between accumulation of MS components and DD.

## Methods

### Participants

The present study design was approved by the Wakayama medical university ethics committee. All participants provided their written informed consent. The present study, entitled the Wakayama Spine Study, was a population-based study of DD performed using a subcohort of the large-scale population-based cohort study called Research on Osteoarthritis/Osteoporosis Against Disability (ROAD). The ROAD study is a nationwide, prospective study of bone and joint diseases consisting of population-based cohorts established in several communities in Japan [19, 20]. A second visit of the ROAD study to the mountainous region of H town and the seacoast region of T town was performed between 2008 and 2010. From inhabitants participating in the second visit of the ROAD study, 1,063 volunteers were recruited for MRI examinations. Among the 1,063 volunteers, 52 declined to attend the examination; therefore, 1,011 inhabitants were recruited for registration in the Wakayama Spine Study. Among the 1,011 participants, those who had an MRI-sensitive implanted device (e.g., pacemaker) or other disqualifiers were excluded. Consequently, 980 individuals underwent whole-spine MRI. One participant who had undergone a previous cervical operation and 4 participants who had undergone previous posterior lumbar fusion were excluded from the analysis. Whole-spine MRI results were available for 975 participants (324 men, 651 women) with an age range of 21 to 97 years (mean, 67.2 years for men, 66.0 years for women). Thirty participants with incomplete anthropometric measurements and 17 participants without blood measurements were excluded. Finally, the present study comprised 928 participants (308 men, 620 women) with a mean age of 67.4 years.

The participants completed an interviewer-administered questionnaire of 400 items that included lifestyle information, such as smoking habit, alcohol consumption, family history, past history, occupation, physical activity, and health-related quality of life. Anthropometric measurements included height, weight, and body mass index (BMI) (weight [kg]/height [m]^2^). An experienced public health nurse measured systolic and diastolic blood pressure (BP) using a mercury sphygmomanometer.

### MRI

A mobile MRI unit (Excelart 1.5 T; Toshiba, Tokyo, Japan) was used in the present study, and whole-spine MRI was performed for all participants on the same day as the questionnaire and anthropometric examination. The participants were supine during MRI, and those with rounded backs used triangular pillows under their head and knees. The imaging protocol included sagittal T2-weighted fast-spin echo (FSE) (repetition time [TR], 4000 ms/echo; echo time [TE], 120 ms; field of view [FOV], 300 × 320 mm) and axial T2-weighted FSE (TR, 4000 ms/echo; TE, 120 ms; FOV, 180 × 180 mm).

Sagittal T2-weighted images were used to assess the intervertebral space from C2/3 to L5/S1. C2/3 to C7/T1, T1/2 to T12/L1, and L1/2 to L5/S1 were defined as the cervical, thoracic, and lumbar region, respectively. Grading of DD was performed by a board certified orthopedic surgeon (M.T.) who was blinded to the background of the subjects. The degree of DD on MRI was classified into 5 grades based on the Pfirrmann system [21], with grades 4 and 5 indicating DD. The signal intensity for grade 4 is intermediate to hypointense to cerebrospinal fluid (dark gray), while the structure is inhomogeneous. The signal intensity for grade 5 is hypointense to cerebrospinal fluid (black), and the structure is likewise inhomogeneous. In addition, the disc space is collapsed. It has been reported that loss of signal intensity is significantly associated with morphologic level of DD and also with water and proteoglycan content in a disc [22]. Therefore, we used a grading system based on signal intensity and disc height.

For evaluating intraobserver variability, 100 randomly selected whole-spine magnetic resonance images were rescored by the same observer (M.T.) more than 1 month after the first reading. Furthermore, to evaluate interobserver variability, 100 other magnetic resonance images were scored by 2 board certified orthopedic surgeons (M.T. and R.K.) using the same classification system. The intra- and interobserver variability for DD, as evaluated by kappa analysis, were 0.94 and 0.94, respectively.

### Blood examination

All blood and urine samples were extracted between 9:00 AM and 3:00 PM. Some samples were extracted under fasting conditions. After centrifugation of the blood samples, sera were immediately placed in dry ice, and transferred to a deep freezer within 24 hours. These samples were stored at –80°C until assayed. For the samples of participants in the baseline study, the following items were measured: blood counts, hemoglobin, hemoglobin A1c (HbA1c), blood sugar, total protein, aspartate aminotransferase, alanine aminotransferase, γ-glutamyl transpeptidase, high-density lipoprotein cholesterol (HDL-C), total cholesterol, triglycerides (TGs), blood urea nitrogen, uric acid, and creatinine. These analyses were performed at the same laboratory within 24 hours after extraction (Osaka Kessei Research Laboratories Inc., Osaka, Japan).

Definitions of MS components were based mainly on the criteria of the Examination Committee of Criteria for MS in Japan [23]. According to the consensus, an abdominal circumference ≥85 cm in men and ≥90 cm in women is a necessary condition for MS. HT was diagnosed as systolic BP ≥130 mm Hg and/or diastolic BP ≥85 mm Hg; DL, as serum TG level ≥150 mg/dL and/or serum HDL-C level <40 mg/dL; and IGT, as fasting serum glucose level ≥100 mg/dL. Recently, the National Cholesterol Education Program’s Adult Treatment Panel III report proposed a new set of criteria to define MS without central obesity, as indicated by waist circumference, as the core feature [24]. Furthermore, compared with BMI, measurement of waist circumference is less reproducible due to lack of uniformity in measurement methods [25, 26]. By contrast, measurement of BMI is more user-friendly and widely practiced. In this study, we decided to use BMI ≥25 kg/m^2^ as an indicator of OW, based on the criteria of the Japan Society for the Study of Obesity [25].

In addition, because not all blood samples were obtained under fasting conditions, we did not use participants’ data concerning serum levels of glucose and TGs because of their large variation depending on hours after eating. Instead, we used serum HDL-C level <40 mg/dL to indicate DL, and serum HbA1c level ≥5.5% to indicate IGT (the value for HbA1c (National Glycohemoglobin Standardization Program (NGSP)) (%) is estimated as an NGSP-equivalent value calculated by the formula HbA1c (%) = HbA1c (Japan Diabetes Society (JDS)) (%) + 0.4%) [27]. These are indices used in the National Health and Nutrition Survey in Japan, which were adopted as criteria for MS in this national screening based on the difficulty of collecting samples under fasting conditions [28].

### Statistical analysis

All statistical analyses were performed using JMP version 8 (SAS Institute Japan, Tokyo, Japan). Differences between the groups depending on the presence or absence of DD were tested using a variance analysis. Multivariable logistic regression analysis was performed to determine the association of OW, HT, DL, and IGT with DD. The DD in the cervical, thoracic, or lumbar region was separately served as an objective variable. Then, to clarify the association between accumulation of MS components and DD, logistic regression analysis was repeated using presence of DD in the cervical, thoracic, and lumbar region, respectively, as the objective variable and number of MS components present as the explanatory variable, after adjusting for age, sex, regional difference, smoking habit, and alcohol consumption. P value of <0.05 was treated as significant.

## Results

Table 1 shows selected characteristics of the participants, including age, height, weight, BMI, systolic and diastolic BP, and serum levels of HDL-C and HbA1c, classified by sex. Table 1 also shows the proportion of subjects who smoked (regularly or more than once a month) and consumed alcohol (regularly or more than once a month), and the prevalence of OW, HT, DL, and IGT. In the total population, the MS component with the highest prevalence was HT, followed by OW, IGT, and DL.

**Table 1:** Background characteristics of the participants.

	Overall	Men	Women
**No. of participants**	**928**	**308**	**620**
**Mean (SD) selected characteristics**			
Age (years)	67.4 (12.3)	68.5 (12.4)	66.8 (12.2)
Height (cm)	155.8 (9.4)	160.5 (8.0)	153.4 (9.1)
Weight (kg)	56.7 (11.5)	60.2 (11.4)	55.0 (11.2)
Body mass index (kg/m^2^)	23.3 (3.6)	23.7 (3.3)	23.1 (3.7)
Systolic BP, mmHg	139.5 (19.6)	141.3 (18.5)	138.7 (20.0)
Diastolic BP, mmHg	76.0 (11.5)	78.1 (12.5)	74.9 (10.9)
Serum levels of HDL-C, mg/dl	63.2 (16.2)	56.0 (14.8)	66.8 (15.7)
Serum levels of HbA1c, %	5.3 (0.7)	5.3 (0.9)	5.2 (0.6)
**Prevalence of selected characteristics, %**			
Smoking habit	10.1	23.3	3.5
Alcohol consumption	31.2	57.5	18.2
**Prevalence of each metabolic abnormality, %**			
Obesity	29.4	32.5	27.9
Hypertension	74.7	78.9	72.6
Dyslipidemia	4.5	10.1	1.8
Impaired glucose tolerance	23.3	27.3	21.3

Values are the means ± standard deviation. HDL-C = high density lipoprotein cholesterol, HbA1c = glycosylated haemoglobin, ABI = ankle brachial index, SD = standard deviation

Table 2 shows the mean value of each MS component according to absence and presence of DD in the cervical, thoracic, and lumbar region, respectively. Mean values of age, BMI, systolic BP, and HbA1c were significantly higher, while those of HDL-C were significantly lower, in subjects with DD than in those without DD.

**Table 2:** Mean value (SD) of each demographic characteristics and measurements in the absence and presence of disc degeneration in the cervical, thoracic, and lumbar region, respectively.

	Cervical			Thoracic			Lumbar		
	Presence of DD	Absence of DD	p-value	Presence of DD	Absence of DD	p-value	Presence of DD	Absence of DD	p-value
**No. of participants**	592	336		578	350		839	89	
**Demographic characteristics and measurements**									
Age (years)	70.8 (11.3)	61.4 (11.7)	0.0001	71.7 (10.3)	60.1 (11.9)	0.0001	67.7 (11.8)	64.0 (16.0)	0.007
Body mass index (kg/m^2^)	23.5 (3.6)	23.0 (3.6)	0.0552	23.6 (3.7)	22.9 (3.3)	0.0058	23.4 (3.6)	22.2 (3.3)	0.0028
Systolic BP, mmHg	141.3 (19.1)	136.6 (19.9)	0.0004	142.5 (18.8)	134.7 (19.8)	0.0001	140.0 (19.8)	134.9 (16.9)	0.0188
Diastolic BP, mmHg	75.5 (11.2)	76.9 (12.1)	0.0663	75.6 (11.2)	76.7 (12.0)	0.1707	76.1 (11.5)	74.9 (12.1)	0.3362
Serum levels of HDL-C, mg/dl	61.8 (15.2)	65.7 (17.5)	0.0004	61.9 (15.4)	65.3 (17.3)	0.0022	63.2 (16.1)	63.5 (17.3)	0.853
Serum levels of HbA1c, %	5.3 (0.7)	5.2 (0.7)	0.0011	5.4 (0.8)	5.1 (0.5)	0.0001	5.3 (0.7)	5.1 (0.5)	0.0067

DD = disc degeneration, BMI = body mass index, BP = blood pressure, HDL-C = high density lipoprotein in cholesterol, HbA1c = hemoglobin A1c, SD = standard deviation

Differences between the groups depending on the presence or absence of DD were tested using a variance analysis.

To determine the associations of DD with OW, HT, DL, and IGT, multivariable logistic regression analysis was performed (Table 3). OW was significantly associated with presence of DD in the cervical, thoracic, and lumbar regions. In addition, HT, DL, and IGT were significantly associated with presence of DD in the thoracic region, but not with DD in the cervical and lumbar regions.

**Table 3:** Association of OW, HT, DL and IGT in cervical, thoracic and lumbar region, respectively.

		Cervical		Thoracic		Lumbar	
		OR (95%CI)	p-value	OR (95%CI)	p-value	OR (95%CI)	p-value
**Overweight**	**Yes vs no**	1.28 (0.92–1.78)	0.1397	1.75 (1.24–2.51) *	0.0016	1.87 (1.06–3.48) *	0.0306
**Hypertension**	**Yes vs no**	1.19 (0.85–1.66)	0.286	1.54 (1.09–2.18) *	0.0138	0.88 (0.52–1.45)	0.6189
**Dyslipidemia**	**Yes vs no**	1.06 (0.49–2.17)	0.8853	0.42 (0.21–0.86) *	0.0176	0.87 (0.34–2.70)	0.7963
**Impaired Glucose Tolerance**	**Yes vs no**	1.27 (0.88–1.85)	0.1943	1.65 (1.12–2.48) *	0.0115	1.48 (0.80–2.95)	0.2211
**Age**	**over 65 vs under 65**	2.98 (2.16–4.11) * * *	<0.0001	5.76 (4.10–8.16) * * *	<0.0001	4.72 (2.47–9.69) * * *	<0.0001
**Sex**	**Women vs men**	1.32 (0.92–1.90)	0.1342	1.02 (0.70–1.48)	0.9198	0.95 (0.56–1.66)	0.8691
**Regional difference**	**Mountainous town vs seacoast town**	1.75 (1.13–2.78) *	0.012	1.20 (0.77–1.89)	0.4353	0.14 (0.07–0.27) * * *	<0.0001
**Smoking habit**	**Yes vs no**	0.88 (0.54–1.44)	0.6002	0.90 (0.54–1.49)	0.6707	0.56 (0.29–1.12)	0.0984
**Alcohol consumption**	**Yes vs no**	0.94 (0.67–1.33)	0.7332	0.96 (0.67–1.37)	0.8332	0.92 (0.55–1.55)	0.7456

Multivariable logistic regression analysis was performed to determine the association of OW, HT, DL, and IGT with DD. The DD in the cervical, thoracic, or lumbar region was separately served as an objective variable. DD = disc degeneration, OW = Overweight, HT = Hypertension, DL = Dyslipidemia, IGT = Impaired Glucose Tolerance. Overweight was diagnosed as BMI ≥ 25, Hypertension was diagnosed as systolic BP ≥ 130 mm Hg and/or diastolic BP ≥ 85 mm Hg, DL was diagnosed as serum HDL-C level < 40 mg/dl, Impaired Glucose Intolerance was diagnosed as serum HbA1c level ≥ 5.5%

OR = odds ratio, 95% CI = 95% confidence interval

*p value < 0.001

***p value < 0.0001

**Fig 1:**
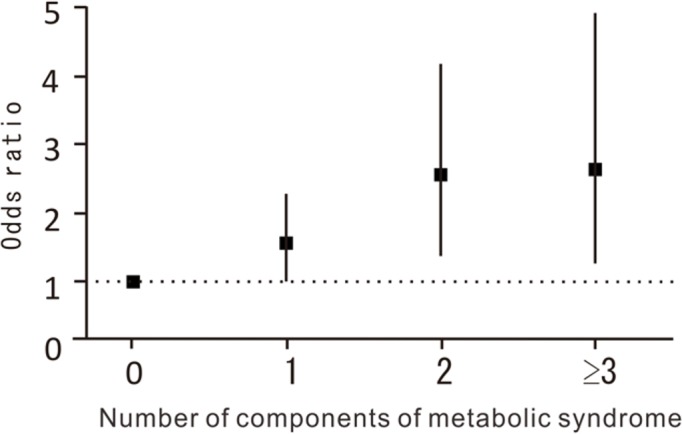
ORs of the number of MS components for the presence of DD in the thoracic region, compared with no components present. Subjects with 1 or more MS components had a higher OR for presence of DD compared with those without MS components.

Next, to determine the effect of accumulation of MS components on DD in the thoracic region, we examined the association of number of MS components present with DD after adjusting for age, sex, regional difference, smoking habit, and alcohol consumption. Fig 1. shows the odds ratio (OR) of number of MS components for presence of DD in the thoracic region. Subjects with 1 or more MS components had a higher OR for presence of DD compared with those without MS components (vs. no component; 1 component: OR, 1.58; 95% confidence interval [CI], 1.03–2.42; p = 0.0353; 2 components: OR, 2.60; 95% CI, 1.61–4.20; p < 0.0001; ≥3 components: OR, 2.62; 95% CI, 1.42–5.00; p = 0.0021).

## Discussion

The present study was the first to determine the associations between MS components and DD in the entire spine using whole-spine MRI in a large population. We elucidated that OW was significantly associated with presence of DD in the entire spine, including the cervical, thoracic, and lumbar regions. HT, DL, and IGT were significantly associated with presence of DD in the thoracic region, but not with DD in the cervical and lumbar regions. Furthermore, we also found that accumulation of MS components was significantly associated with presence of DD in the thoracic region.

Regarding the association between degenerative musculoskeletal disease and metabolic risk factors, Yoshimura et al. clarified the association between accumulation of metabolic risk factors and presence and occurrence of knee osteoarthritis (OA) [29, 30]. Hart et al. found that metabolic risk factors, such as high blood glucose level, hypercholesterolemia, and even treated HT, were associated with development of knee OA [31]. Furthermore, Anekstein et al. clarified the association between diabetes mellitus and lumbar spinal stenosis in the patients [32]. However, to our knowledge, there has been no report concerning the association between MS components and DD in the spine, especially the entire spine, using whole-spine MRI in a large population.

In the present study, OW was significantly associated with presence of DD in the cervical, thoracic, and lumbar regions. The association between OW and DD has been previously reported, and Liuke et al. found that past OW was more strongly associated with DD than present OW [13]. Samartzis et al. reported that DD in the lumbar region was significantly associated with OW and obesity [14]. On the other hand, according to Okada et al. and Matsumoto et al., DD in the cervical and thoracic regions did not have significant correlation with BMI [32, 33]. Therefore, the association remains controversial. The present study is the first to determine the association of OW with DD in the entire spine using a population-based design, and found that OW was significantly associated with DD in not only the lumbar region but also the cervical and thoracic regions. DD is influenced by inflammatory cytokines, such as adipokines, known as key metabolism mediators [34–37]. Inflammatory cytokines, such as leptin, adiponectin, and resistin, have more addressed in body fat [34, 38]. Thus, OW may lead to an increase in adipokine secretion of proinflammatory cytokines and metabolic mediators; thus, all intervertebral discs in the entire spine may be influenced by inflammatory cytokines. Further research is needed to elucidate the mechanism through which OW affects DD since both direct mechanical stress and indirect factors affect the intervertebral discs.

To our knowledge, there has been less report regarding the association of HT with DD or lumbar spinal stenosis as spinal disorder [9, 39]. HT is a well-known risk factor for development of atherosclerosis [40]. Thus, HT might lead to vascular insufficiency to the disc, due to atherosclerosis, which can affect nutrient and metabolite transport into the disc.

The present study also confirmed the significant association between IGT and DD. In one study, it was reported that diabetic sand rats had more dehydrated discs compared with a control group [17]. In the Nurses’ Health Study, IGT increased the risk of lumbar disc herniation [41]. However, several previous reports on DD also showed a weak association with IGT [9, 16, 42]; this may be due to their investigation of DD in only the lumbar region. In this study, we found an association between IGT and presence of DD in the thoracic region. Therefore, IGT, which is well known for causing microangiopathy throughout the whole body, also might be a predisposing factor for development of DD. Furthermore, advanced glycation end products accumulate in the intervertebral discs with aging, particularly when the concentration of serum glucose is high, such as in IGT [43]. Therefore, IGT might be associated with DD.

In this study, we found a negative association between DL and DD. The association of DL and DD also remains controversial in previous reports [9, 18, 44]. We believe that DD might be the result of decreased blood supply, caused by DL, to the already poorly vascularized discs [45, 46]. The mean HDL-C was higher in women than in men, as shown in Table 2. Because women in Japan use health services more frequently compared with men [28], the proportion of patients with DL in women was higher than that in men. This might have influenced the negative association between DD and DL. In a follow-up study, we will further investigate the association between DL and DD.

We found no associations of HT, DL, and IGT with DD in the cervical and lumbar regions. Since the cervical and lumbar regions comprise mobile segments, the intervertebral discs are easily affected by mechanical and motion stress, while the effect of endogenous factors might be masked. In contrast, in the thoracic region, mechanical stress on the intervertebral discs is reduced because the region is stabilized by the thoracic cage. Distinct associations among DD in the cervical, thoracic, and lumbar regions might indicate the effects of HT, DL, and IGT on DD are due to endogenous factors. To clarify risk factors for DD, particularly endogenous risk factors, it may be useful to examine associations in not only the cervical and lumbar regions, but in the thoracic region as well.

### Study limitations

This study has several limitations. First, this was a cross-sectional study; thus, the causal relationships between MS components and DD remain unclear. These can only be ascertained by a follow-up study that clarifies the incidence and/or progression rates of DD in the same cohort. Second, the participants included in the present study may not represent the general population since they were recruited from only 2 local areas. To confirm whether the participants are representative of the Japanese population, we compared anthropometric measurements and frequencies of smoking and alcohol consumption between the general Japanese population and the study participants. No significant difference in BMI was observed (men: 24.0 kg/m^2^ vs. 23.7 kg/m^2^, p = 0.33; women: 23.5 kg/m^2^ vs. 23.1 kg/m^2^, p = 0.07). Further, the proportion of men who smoked and who consumed alcohol (those who regularly smoked or consumed alcohol more than once per month) and the proportion of women who consumed alcohol were significantly higher in the general Japanese population than in the study population, whereas there was no significant difference in the proportion of women who smoked (men who smoked: 32.6% vs. 23.3%, p = 0.015; women who smoked: 4.9% vs. 3.5%, p = 0.50; men who consumed alcohol: 73.9% vs. 57.5%, p < 0.0001; women who consumed alcohol: 28.1% vs. 18.2%, p < 0.0001). These results suggest the likelihood that, in this study, participants had healthier lifestyles than those of the general Japanese population [28]. This “healthy” selection bias should be taken into consideration when generalizing the results obtained from the Wakayama Spine Study. In addition, since the blood samples obtained were not always from participants under fasting conditions, we used serum HDL-C level <40 mg/dL, and not TG level, to indicate DL, and serum HbA1c level ≥5.5%, and not blood glucose level, to indicate IGT, which are indices used by the National Health and Nutrition Survey in Japan [28]. These differences in the definition of MS might have skewed the true association between MS and DD.

## Conclusions

We investigated the associations between MS components and DD in the cervical, thoracic, and lumbar regions in a large population of individuals ranging in age from 21 to 97 years. We revealed that OW was significantly associated with presence of DD in the entire spine, and that HT and IGT were significantly associated with presence of DD in the thoracic region. We also found that subjects with 1 or more MS components had a higher OR for presence of DD compared with those without MS components. The prevention of MS may be useful for avoiding DD. Further investigations, along with continued longitudinal surveys of the Wakayama Spine Study, will elucidate the associations between MS components and occurrence or progression of DD.

## Abbreviations

DD: disc degeneration
MS: metabolic syndrome
OW: overweight
HT: hypertension
DL: dyslipidemia
IGT: impaired glucose tolerance
ROAD: Research on Osteoarthritis/Osteoporosis Against Disability
